# The Americans with Disabilities Act and Equal Access to Public Spaces

**DOI:** 10.3390/laws13010005

**Published:** 2024

**Authors:** Barry A. Whaley, Jonathan G. Martinis, Giuseppe F. Pagano, Sara Barthol, Jessica Senzer, Pamela R. Williamson, Peter D. Blanck

**Affiliations:** Burton Blatt Institute, Syracuse University, Syracuse, NY 13244-2130, USA

**Keywords:** accessibility, disability rights, disability law, discrimination, public spaces

## Abstract

Since the passage of the landmark Americans with Disabilities Act of 1990, the United States federal government, states, and localities have passed laws and created policies intended to ensure that people with disabilities had full and equal access to public spaces. Nevertheless, more than three decades after the ADA, people with disabilities continue to face architectural and other barriers to community inclusion and participation. This article describes laws, policies, and initiatives that are implemented in the United States at the federal, state, and local levels to address these barriers, examines their effectiveness, and describes the views of advocates working in furtherance of the rights of people with disabilities and the inclusiveness of public spaces. We conclude by providing brief recommendations for ways federal, state, and local governments may ensure people with disabilities have full and equal access to public spaces.

## 1. Introduction

The United States of America was founded upon the promise of equal rights to “Life, Liberty, and the Pursuit of Happiness” for all. Nevertheless, it took 214 years—until passage of the landmark Americans with Disabilities Act of 1990 (ADA)—for people with disabilities to be finally and fully included in the “all”. The ADA unequivocally states people with disabilities do and should have the “right to fully participate in all aspects of society”, while recognizing they are historically and still systematically excluded from such participation by barriers including “the discriminatory effects of architectural … barriers, … [and] failure to make modifications to existing facilities” (Americans with Disabilities Act of 1990, sec. 12101(a)).

In the U.S., one in every four adults has a disability as defined by the ADA (Centers for Disease Control and Prevention 2020) and 11.1% of Americans with disabilities have mobility impairments that limit their ability to walk or climb stairs (ibid.). The sweeping scope and aims of the ADA, like other laws before it, including Section 504 of the Rehabilitation Act of 1973 seeks to address and ameliorate the many ways discrimination negatively impacts the everyday lives and opportunities of this substantial segment of the U.S. population (Rehabilitation Act of 1973, sec. 504).

There are five sections, or Titles, to the ADA. This article focuses on Title II which, among other things, prohibits state and local governments from denying or excluding people with disabilities equal access to their services, programs, and activities due to inaccessible or unusable facilities (Americans with Disabilities Act of 1990; Jones 2011). Regulations to the ADA define “facility” broadly to include “walks, passageways … or other real or personal property, including the site where the building, property, structure, or equipment is located” (Americans with Disabilities Act of 1990). Nonetheless, more than three decades after the passage of the ADA, too many public facilities—including basic accommodations like sidewalks—remain inaccessible, with progress toward the promise of the law often being stalled by disagreements over who should make and pay for modifications to existing and new facilities necessary to ensure accessibility (Jones 2011).

In this article, we first summarize federal statutes, regulations, and case law regarding accessibility of public transportation, sidewalks, and Rights-of-Way (public spaces). We then review state and local laws in New York and Georgia that impact accessibility of public spaces and review efforts made in those states to ensure accessibility. We then provide first-person accounts of advocates with expertise and experience in working on matters related to the accessibility of public spaces. We conclude by offering recommendations for ways federal, state, and local governments may address the continuing problem of inaccessible public spaces and ensure people with disabilities are truly able to “fully participate in all aspects of society”.

## 2. The Role of the Burton Blatt Institute in the Inclusive Public Space Project

This article, and the research and work leading up to it, is part of the Inclusive Public Space Project (University of Leeds n.d.) (IPS Project). Since January 2019, the IPS Project has been investigating the negative impacts of exclusionary, inaccessible public space design and maintenance. The project focuses on how unequal access to city streets impacts people with disabilities, older adults, children, parents, and caregivers in the United Kingdom, India, Kenya, the Netherlands, and the United States.

In the United States, the Burton Blatt Institute (BBI) at Syracuse University has collaborated with IPS Project researchers to investigate how federal, state, and local laws addressed and impacted the accessibility of public spaces. We also conducted more in-depth reviews of law, policy, and activity addressing the accessibility of public spaces in the cities of Syracuse, New York, and Atlanta, Georgia.

Syracuse was chosen for a deeper review because it was the location of Syracuse University, home of the Burton Blatt Institute. More importantly, Syracuse, a small city established 200 years ago, has a declining population and the number of residents living in poverty is 2.5 times the national average, resulting in a smaller tax base and fewer dollars for infrastructure improvements (Rudd 2023). The city is also the snowiest in the United States due to its proximity to Lake Ontario. Syracuse’s 127.8 average inches of snowfall creates a challenge for traversing city sidewalks, especially for people with disabilities and therefore, a concomitant need to ensure sidewalks are clear and accessible (Donegan 2022).

Atlanta is the eighth-largest metropolitan area in the United States (Williams 2023). Atlanta was chosen for a deeper review due to the city’s 2009 settlement agreement with the U.S. Department of Justice and its aftermath. The settlement sought to ensure full and equal access to citizens with disabilities by requiring the city to comply with the ADA (U.S. Department of Justice 2009). Nine years after the settlement agreement, a class action suit was filed on behalf of people with disabilities claiming the city continued to neglect deteriorating sidewalks, curb ramps, and pedestrian rights of way (Radford Scott LLP n.d.).

We began our work by examining how federal laws like the ADA, Section 504, the Federal-Aid Highway Act, and the Urban Mass Transportation Act established minimum standards to ensure accessible public spaces. We examined how the Access Board Public Rights-of-Way Guidelines, first proposed in July 2011, and nearing final adoption in 2024, provided guidance on best practices to improve accessibility in public spaces. We also examined how laws like the Fixing America’s Surface Transportation (FAST) Act used federal funding and grants to influence the accessibility of public spaces (Fixing America’s Surface Transportation Act of 2015). Finally, we examined state and local laws in New York and Georgia relevant to the accessibility of public spaces and how those laws interacted with the U.S. federal framework.

Our work is consistent with and furthers the aims of the larger IPS Project in that it seeks a deeper understanding of: (1) how existing laws and policies impact the accessibility of public spaces; (2) how and why accessing public spaces is challenging for people with disabilities; and (3) how people with disabilities and governments do or can use existing laws and policies to ensure public spaces are and remain accessible.

We conducted our review with the mindset that civil rights laws like the ADA were, and must be, premised on equal access in fact, not just equality in theory; that legislation, in and of itself, was of little use without comprehensive efforts to comply and ensure compliance with it. Most relevant to this project, we acknowledge the right to access public spaces is often a determinant of other rights: people cannot go to work, appear at court, or shop in a store if they cannot access public transportation, sidewalks, and rights of way leading to those places.

Accordingly, our work also examined policies, court cases, and other efforts to interpret, implement, and enforce federal, state, and local civil rights laws. In addition to the detailed law and policy review we carried out as part of the IPS Project, we helped recruit disabled pedestrian participants who were interviewed by researchers at the University of Leeds about their experiences of exclusion. We also worked with IPS Project staff to identify and recruit stakeholder participants in the United States with relevant legal, policy, or advocacy experience. Twenty-two stakeholder participants were interviewed online in either small focus groups or one-to-one interviews by researchers based at the University of Leeds. This group was comprised of eight attorneys; eight stakeholders who engaged in advocacy work; and six stakeholders who worked in policy formulation or implementation. For purposes of this article, we draw on our law and policy review and data from interviews of stakeholders with legal experience and expertise on matters relating to the accessibility of public spaces at the city, state, or federal level.

## 3. U.S. Federal Laws and Policies Addressing Accessibility of Public Spaces

The disability rights movement in the United States began in earnest in the 1960s, when people with disabilities began demanding equal access to public services and spaces. Direct and targeted advocacy by people with disabilities led to the passage and implementation of Section 504 (Rehabilitation Act of 1973, sec. 504), which required people with disabilities to have equal access to programs and services that received federal funds (e.g., Blanck 2020).

Regulations to Section 504 set forth accessibility standards and legal requirements that federal fund recipients, including public transportation providers, must meet (e.g., Blanck 2020). As such, Section 504 also bolstered the intent and impact of similar laws setting forth accessibility standards and legal requirements designed to ensure people with disabilities had full and equal access to public transportation, sidewalks, and Rights-of-Way, including the Urban Mass Transportation Act, Federal-Aid Highway Act, and the Air Carrier Access Act (e.g., Blanck 2020).

Building upon these laws, the ADA guarantees equal access to people with disabilities in all facets of life and spheres of society. The ADA aims to address and ameliorate physical, systemic, attitudinal, and other problems that leave people with disabilities vulnerable to discrimination, recognizing people with disabilities have historically experienced discrimination in public and private services because of “outright intentional exclusion, the discriminatory effects of architectural, transportation, and communication barriers, overprotective rules and policies, failure to make modifications to existing facilities and practices, exclusionary qualification standards and criteria, segregation, and relegation to lesser services, programs, activities, benefits, jobs, or other opportunities” (Americans with Disabilities Act of 1990, sec. 12101). Title II of the ADA requires state and local governments to ensure people with disabilities have equal access to public programs and services. ADA regulations also specify state and local governments must ensure people with disabilities are able to access public spaces and transportation (ibid. sec. 35101).

The ADA was unprecedented in intent and scope. However, while the ADA required people with disabilities to have full and equal access to public services and spaces, it fell to public policymakers and agencies to develop specific requirements and standards—to definitively say what accessibility was and what public and private entities must do to ensure it (e.g., Blanck 2020). Consequently, several federal agencies have promulgated regulations and policies designed to flesh out the ADA’s requirements and ensure people with disabilities had full and equal access to public spaces.

In the United States, administrative agencies have the authority to propose regulations that are within the scope of their statutory authority. Each year an agency may publish an Agenda of Regulatory and Deregulatory Actions that announces future rule-making activities. If it does, it must next publish an Advance Notice of Proposed Rulemaking in the Federal Register, a daily publication of Presidential documents and new and amended regulations. Agencies must then allow the public to review and comment on its proposed rule(s). An agency may also hold public hearings on proposed rules. The agency then publishes its Final Rule and begins a compliance, interpretation, and review phase that allows industries affected by the rule time to understand the new regulatory requirements. Affected federal agencies must then adopt the new regulations before the regulations become law (Federal Register n.d.).

Following this process, the U.S. Access Board developed the ADA Accessibility Guidelines for Buildings and Facilities, which set forth specific standards that pathways, door-ways, facilities, and other architectural features in public and private accommodations must meet to comply with the ADA. The guidelines have been incorporated into the ADA, and therefore are legally enforceable, through regulations promulgated by the U.S. Department of Justice (Rothstein 2014). The Department of Justice’s ADA regulations also require streets, roads, and highways must “contain curb ramps or other sloped areas” to ensure access for people with disabilities (Americans with Disabilities Act of 1990 sec. 35.151(i)(1)).

The Access Board also developed Public Rights-of-Way Guidelines (“PROWAG”) addressing accessibility in public spaces and Rights-of-Way. These include requirements for detectable warning surfaces on curb ramps and blended transitions at street crossings intended for pedestrian use; accessible and audible signals and pedestrian pushbuttons; and pedestrian-activated signals at roundabout intersections with multi-lane pedestrian street crossings (American Association of People with Disabilities 2020a, 2020b). In 2023, the U.S. Access Board issued a final rule on the PROWAG guidelines. The document provides technical guidance for various spaces and elements in the public right-of-way including pedestrian access routes, standards for pedestrian signals, curb ramps, and street furniture. These guidelines become law, and enforceable as part of the ADA, once adopted by the U.S. Department of Justice and the Department of Transportation (U.S. Access Board 2023).

Similarly, the U.S. Department of Transportation developed standards based on the U.S. Access Board’s ADA Accessibility Guidelines (ADAAG). These standards applied to state and local public transportation systems, specifically bus and railway stops and stations, as well as public transportation vehicles and facilities (U.S. Access Board n.d.). The Department also requires state and local governments to maintain equipment and features of facilities that provide people with disabilities with access, including sidewalks that may require snow removal and other maintenance (U.S. Department of Transportation 2023).

The U.S. federal government can, and often does, use funding to ensure compliance with nondiscrimination laws like the ADA and Section 504. While the Eleventh Amendment to the U.S. Constitution (U.S. Const. amend. XI) holds “nonconsenting States may not be sued by private individuals in federal court” (Bd. of Trs. of the Univ. of Ala. v. Garrett, 531 U.S. 356, 363 (2001)), that immunity is “not absolute” (Garcia v. S.U.N.Y. Health Scis. Ctr. of Brooklyn, 280 F.3d 98, 108 (2d Cir. 2001)). As the *Garcia* Court held, “when providing funds from the federal purse, Congress may require as a condition of accepting those funds that a state agree to waive its sovereign immunity from suit in federal court.” (Garcia at 113).

Section 504 includes such a waiver (*Americans with.Disabilities Act of 1990*, sec. 2000d-7) authorizing people with disabilities to sue state and local governments when they are excluded from participation or denied the benefits of a “program or activity receiving Federal financial assistance” (*Rehabilitation Act of 1973*, sec. 794(a)). “Program or activity” means “*all* of the operations of a department, agency, special purpose district, or other instrumentality of a State or of a local government … any part of which is extended Federal financial assistance.” (*Rehabilitation Act of 1973*, sec 794(b) (emphasis added)). Regulations to Section 504 define “recipient” of federal funding as “any instrumentality of a state … to which Federal financial assistance is extended directly or through another recipient” (45 C.F.R. sec. 84.3(f)).

Accordingly, when state and local governments receive federal funding to provide or maintain public spaces, they are subject to the nondiscrimination requirements of Section 504. As a result, people with disabilities may enforce those requirements by filing lawsuits against such government entities if public spaces are not designed, built, or maintained in an accessible condition.

Other federal funding mechanisms expressly stated projects funded with federal dollars must be accessible to people with disabilities. For example, the Fixing America’s Surface Transportation (FAST) Act authorizes all U.S. highway and transit federal funding. FAST specifically requires recipients of such funding ensure sidewalks, transportation, and other transportation projects ensure accessibility for people with disabilities (Fixing America’s Surface Transportation Act of 2015).

## 4. Representative U.S. Case Law Addressing Accessibility of Public Spaces

The interpretation, implementation, and enforcement of the ADA and similar nondiscrimination laws is shaped by case law. Unfortunately, existing case law illustrates the challenges being faced by Americans with disabilities who file lawsuits seeking to ensure the accessibility of public spaces.

Two cases are particularly instructive. In *Culvahouse v. City of LaPorte*, two plaintiffs filed a lawsuit against the city of LaPorte, Indiana alleging the city’s sidewalks were in bad repair, which made use of those sidewalks by persons with disabilities difficult if not impossible, in violation of the ADA. Both plaintiffs claimed the ADA violations prevented them from taking part in community activities.

A survey identified the work needed to make the sidewalks accessible, leading to disputes as to who was responsible for making and paying for the needed renovations. The plaintiffs argued the city was responsible. The city countered its sidewalks were not a “service, program, or activity” under the ADA and, if they were, then individual property owners were required to make and pay for any renovations to them. Finally, the city argued even if it was required to maintain the sidewalks, the cost of doing so would be an “undue burden”.

The Court ruled in favor of the plaintiffs, holding LaPorte’s sidewalks were a “service, program, or activity” for purposes of the ADA. The Court also held the city was responsible, under Indiana Law, for maintaining sidewalks and, therefore, was required to make and pay for the renovations needed to ensure the sidewalks complied with the ADA. Finally, the Court held the city did not prove paying for the renovations was an “undue burden” (Culvahouse v. City of LaPorte, 679 F. Supp. 2d 931 (2009)).

In *Cohen v. City of Culver City*, the plaintiff sued the city of Culver City, California, and others, alleging violations of the ADA and various California statutes. Mr. Cohen alleged he was walking through an outdoor car show on public streets and a vendor’s displays blocked the curb ramp providing access to the sidewalk in front of Mr. Cohen’s hotel. Mr. Cohen then attempted to walk around the display to step up onto the sidewalk but fell and was injured.

After the trial court granted the city’s motion for summary judgement, the case went before the United States Court of Appeals for the Ninth Circuit. The plaintiff argued the city had violated its obligations under the ADA by allowing the vendor’s display to block the curb ramp, impeding safe access to the public sidewalk for pedestrians with disabilities. The city countered it provided a marginally longer alternative route for people with disabilities and, therefore, did not violate the ADA.

The Court of Appeals reversed the trial court’s decision. The Court held the city violated state and federal laws because its sidewalks were initially ADA-compliant and became out of compliance through poor oversight and planning of the car show event. The Court further held the city did not excuse its violation by providing a longer alternative route (Cohen v. City of Culver City, 754 F 3d 690 (2014)).

*Culverhouse* and *Cohen* demonstrate the difficulties people with disabilities face when attempting to ensure public spaces are accessible to them. In both cases, the city defendants raised factual and procedural arguments that had nothing to do with whether their sidewalks were accessible to people with disabilities—such as the *Culverhouse* defendant claiming renovating the sidewalks to be in compliance with the ADA would be an “undue burden” and the *Cohen* defendant’s argument that it was not responsible for the violation because there was another, longer, route the plaintiff could have taken. The cases demonstrate, even when their public spaces are inaccessible, state, and local governments have many avenues they can use to attempt to avoid complying with the ADA.

## 5. State and Local Law and Policy in New York and Georgia Addressing Accessible Public Spaces

### 5.1 New York and Georgia State Law and Policy

In the U.S., each state has the power to pass and enforce its own laws, provided they do not conflict or interfere with federal law. In this section, we examine how New York and Georgia state law and policy impact people with disabilities’ rights and opportunities to access public spaces.

While New York does not have a separate law addressing the rights of people with disabilities, it has anti-discrimination laws applicable to people with disabilities. The New York State Human Rights Law provides every citizen with “an equal opportunity to enjoy a full and productive life” and prohibits discrimination based on several characteristics, including disability (New York State Division of Human Rights 2021). The law uses a similar framework as the ADA—requiring reasonable modifications to policy and practice and the removal of communication and architectural barriers—to ensure equal opportunities for people with disabilities (Blanck et al. 2021).

In addition, New York State law requires public streets and sidewalks adjacent to curbs, parking lots, and roads and highways open to traffic to be constructed or renovated in ways that allow reasonable access for pedestrians with disabilities and comply with ADA accessibility guidelines (NY High. Law sec. 330). The state has also adopted the PROWAG standards requiring accessibility in public sidewalks and Rights-of-Way and required state, county, and local authorities to consider the mobility of all users as they developed transportation projects (New York State Department of Transportation 2016).

According to New York’s most recent ADA Transition Plan, these and other efforts have resulted in 82 percent of state-owned sidewalks and 66 percent of curb ramps meeting ADA requirements (New York State Department of Transportation 2016).

Like New York, Georgia does not have a specific state law addressing and ensuring the rights of people with disabilities. However, the state has an ADA coordinator to “provide comprehensive educational and technical support for State agencies so that those programs, services, and activities being operated by the State of Georgia are accessible and usable by everyone … (and) to maintain compliance with the Americans with Disabilities Act” (Georgia State ADA Coordinator’s Office 2020).

In addition, the Georgia Department of Transportation has adopted and developed policies designed to increase the accessibility of public spaces. The Department has developed minimum standards for accessibility in construction projects that were based on federal law and regulations including the ADA and PROWAG (Georgia Department of Transportation 2023).

### 5.2. County Law and Policy in New York and Georgia

Counties in New York and Georgia have their own laws and policies designed to ensure equality and inclusion in public services and spaces. In this section, we review such laws and policies in Onondaga County, New York and Fulton County, Georgia, the counties containing Syracuse and Atlanta, respectively.

In New York, the Onondaga County Code mandates sidewalks in the county be cleared of snow and ice (Onondaga County Code sec. 253–1L) However, according to New York State law, property owners, and not the county, have the obligation to maintain sidewalks in accessible condition (NYS Property Maintenance Code 302).

The Onondaga County Code also stated the county must support “services as may be required for the construction, repair, alteration, and demolition of all … facilities in the nature of public works within county jurisdiction or where contractually or otherwise appropriate or lawful and where not otherwise specifically assigned in the chart of this code.” The Code charged a Deputy Commissioner of the county’s Department of Transportation to oversee the construction, maintenance, repair, supervision, alteration, demolition, custodial care of, snow removal from highways, and other Rights-of-Way owned by the county (Onondaga County Code sec. 20.02).

Onondaga County has a Commission on Human Rights, which oversees the county’s anti-discrimination policies, including those addressing the rights of people with disabilities (Onondaga County Commission on Human Rights 2021a). The county also employs an ADA coordinator and a human rights council that can address and resolve accessibility concerns (Onondaga County Commission on Human Rights 2021b). Finally, the county hosts the Central New York Transportation Authority, which operates the Call-A-Bus service to provide public transportation to people with disabilities who cannot access traditional public transportation (PEACE Inc 2020).

In Georgia, Fulton County’s law enhances the accessibility of public sidewalks and rights of way by setting forth specific requirements for curb cuts in commercial and residential areas (Fulton County Code 1983, sec. 28-2-42). For example, the code requires all curb cuts align with the edge of the pavement and be constructed with a “five-foot flare as measured along the curbline of the street” (Fulton County Code 1983, sec. 62–76).

Fulton County has also created a multi-phase Self Evaluation and Transition Plan designed to ensure the county complied with the ADA (Fulton County 2021). The Plan states the county may not prevent people with disabilities from participating in county services, programs, or activities and must ensure its facilities are accessible to people with disabilities (Fulton County 2021). In addition, Fulton County residents, as well as those in DeKalb County, who could not access public transportation offered by the Metropolitan Atlanta Rapid Transit Authority may use paratransit through the Empowerline program (Equal Opportunity and Nondiscrimination 2021).

### 5.3. City Law and Policy in New York and Georgia

Like states and counties, city municipalities may pass and enforce their own laws and regulations, so long as they do not conflict with federal law. In this section, we discuss laws and policies in the cities of Syracuse and Atlanta that impact people with disabilities’ right and opportunities to access public spaces.

Syracuse has laws protecting people with disabilities’ right to access public sidewalks. The city will require new and renovated sidewalks to be “constructed in a manner that will facilitate use by physically handicapped persons” and explicitly will require curb cuts at intersections and crosswalks to have accessible slip gradients (City of Syracuse’s Code of Ordinances n.d., chap. 24). In addition, the city requires property owners and occupants to maintain the sidewalks that surround their property and states that landowners must not allow their land “to be at any time other than in good repair and in a good and safe condition”. In the winter, property owners or occupants are responsible for ensuring their sidewalks are clear (City of Syracuse’s Code of Ordinances n.d., chap. 24). Finally, Syracuse has adopted a Comprehensive Plan which aimed to identify and implement a year-round solution to maintain sidewalks that were accessible to all (City of Syracuse 2021).

Atlanta has had a more difficult journey toward accessibility. In December 2009, the U.S. Department of Justice entered into a settlement agreement with the city to improve all aspects of civic life for people with disabilities. The settlement agreement called for Atlanta to survey all municipal facilities and programs and make modifications to parking, entrances, paths of travel, parking lots, restrooms, service counters, and drinking fountains to ensure accessibility. The city was also required to create and implement a grievance procedure to address disability discrimination complaints (U.S. Department of Justice 2009).

In the fourteen years since the settlement, Atlanta has failed to address the backlog of sidewalks and ramps in disrepair. One survey found that only twenty percent of the City’s sidewalks were accessible to wheelchair users or motorized scooters, and only roughly thirty percent of sidewalks had curb ramps. (O’Hagan 2021). Atlanta’s continuing failure to ensure accessibility resulted in a class action suit being filed against the city, asking the Court to order the city to remedy its ADA violation (Radford Scott LLP n.d.)

Atlanta has created a “Complete Street Policy”, which aimed to ensure universal accessibility and specifically mentions addressing the accessibility of public Rights-of-Way (City of Atlanta (City of Atlanta 2017–2021) Capital Improvements Program and Community Work Program). Even so, the city’s efforts to improve infrastructure and accessibility have deteriorated in recent years. In 2015 and 2016, voters supported efforts to invest more than $500 million to fund transportation projects and other infrastructure improvements. However, the funding was not enough to complete the planned projects including street improvements, roadway improvements, sidewalk and mobility improvements, and traffic control devices (Renew Atlanta n.d.). This led the city to “drop the $40 million for sidewalk upgrades and reduce the $35 million originally meant for curb cuts to $5 million” (Renew Atlanta n.d.).

## 6. Legal Stakeholders’ Experiences of Inaccessible Public Spaces

As mentioned above, as part of the IPS Project, researchers interviewed stakeholders with relevant legal expertise about their experiences working on matters relating to the accessibility of public spaces at the city, state, or federal level. These stakeholders described experiences and views which echoed the difficulties faced by the plaintiffs in *Culvahouse* and *Cohen*.

One stakeholder remarked:

[C]urb ramps tend to be in most cities more pressing a concern than the sidewalks. Although some cities like Baltimore, the sidewalks are so awful you can’t even get from curb ramp to curb ramp. So that is terrible. And in fact, it’s interesting, there usually is a disability rights conference in Baltimore every year … But many of us have travelled there several years in a row and walked the streets ourselves and been like, what is happening here? What is wrong with this … because there will be just sidewalks that have obviously not been maintained well. (Felicia (2022), attorney)

Another stakeholder expressed concerns about sidewalks not being maintained properly:

I think probably the most pressing is sort of like the condition of the sidewalks themselves. So, one is the, you know, even if you construct a perfectly level sidewalk, over time it’s going to start buckling. And if you’ve got trees, the tree roots can cause them to buckle and so it can make it effectively impassable for somebody who uses a wheelchair and difficult for somebody using like a walker or a cane or that just has other difficulty navigating that kind of unevenness. (Megan (2022), attorney)

Another interviewee reported cities often neglected their responsibility to keep sidewalks and Rights-of-Way in good repair, stating:

There’s [*sic*] issues over who is responsible for the maintenance and ownership of the sidewalk and the intersections because—and the city of Atlanta, they’ve passed statutes saying that the adjacent property owner is responsible. So, we have been arguing well the ADA is a non-delegable duty blah blah blah, but it keeps coming up. You’ve got other utility groups and things like that that have easements so they—the city keeps saying, well we can’t stop Georgia Power from putting a power pole in the middle of the sidewalk, you know. We keep running into that issue. (Adelyn (2022), attorney)

These interviews were conducted with people located in different geographic regions of the country, demonstrating the ongoing problem of inaccessible public spaces was not limited to certain cities or regions but was a nationwide issue that created unequal access and a loss of opportunity for millions of people with disabilities.

## 7. Potential Solutions to Improve the Accessibility of Public Spaces

Our work highlights the need for concerted and comprehensive action to ensure accessibility of public spaces. More than three decades after the ADA and fifty years after Section 504 promised people with disabilities the right to access public spaces, implementation, and enforcement of these laws lagged their intent. As is shown above, even a state considered “progressive” like New York cannot guarantee a person with disabilities is able to access and travel along its sidewalks. Therefore, in this section, we provide recommendations for ways U.S. federal, state, and local governments may keep the promise of equal access and opportunity for all their citizens.

First and foremost, the U.S. federal government must play a lead role in protecting people with disabilities’ hard-won and long-delayed rights. Both the ADA and Section 504 give the federal government the power to pursue litigation against public and private entities that do not comply with those laws (Americans with Disabilities Act of 1990 sec. 12117; *Rehabilitation Act of 1973* sec. 794a). Robust enforcement of these laws, up to and including seeking civil penalties or even withholding federal funding, must be considered when states or localities fail to meet or ignore their legal responsibilities.

For example, the U.S. Department of Justice’s Project Civic Access was designed to identify and address barriers to accessibility in counties, cities, towns, and villages; the project played a lead role in ferreting out Atlanta’s ADA noncompliance leading to a settlement agreement with the city (United States Department of Justice Civil Rights Division 2023). However, in recent years, the project has been less active (ibid.). Reenergizing programs like Project Civic Access will not only increase accessibility in the municipalities they work with, but will also help raise the visibility and re-center the importance of accessible public spaces throughout the United States.

Furthermore, states and their municipalities must take immediate and tangible steps to ensure their public spaces were accessible to people with disabilities—which has been legally required for three decades. While we understand renovations can be costly, there are low-cost options to improve accessibility for people with disabilities. One solution is increasing public education for both individuals and covered entities regarding their rights and responsibilities to make the built environment accessible. Easy-to-understand accessibility checklists are a simple and effective way to engage and educate the public about accessibility and address ADA noncompliance. For example, the Safe Routes Partnership in Georgia provides people with guidance on what to look for when evaluating ADA compliance of sidewalks and other Rights-of-Way. The Partnership also provides information to people with disabilities on how they may report or file complaints regarding inaccessible public spaces (Safe Routes Partnership n.d.). This method of empowering people with disabilities with the knowledge and tools to investigate whether public spaces are accessible, and act if they are not, aligns with the “nothing about us without us” ethos of the disability rights movement (United Nations Enable 2004).

States may also incentivize compliance by publicizing communities that have achieved accessibility and highlighting options to fund necessary renovations. For example, New York resolved a lawsuit alleging the New York City subway system was inaccessible to people with disabilities by accessing funding from the Federal Transit Federal Transit Administration (2021) to renovate subway stations (NY.gov 2023). The state then announced the renovations were complete in July, designated as Disability Pride Month, which focused attention on the need for equal access and accessibility in all public spaces and areas of life (NY.gov 2023; Mass Transit 2023).

Several states have followed this model, and all should—hopefully without first being sued. For example, in 2022, the U.S. government awarded over $680 million in funding to nine states to make their public transportation systems and stations accessible to people with disabilities. In announcing the awards, the U.S. Department of Transportation highlighted both the urgent need to ensure public transportation and spaces were accessible to people with disabilities and how much more needed to be carried out to achieve that goal, stating: “Every day, millions of people rely on our public transit system to get to work, buy groceries, and see their loved ones—yet today, three decades after passage of the Americans with Disabilities Act, hundreds of transit stations are still inaccessible for travelers with disabilities” (U.S. Department of Transportation 2022).

## 8. Conclusions

The ADA, and federal, state, and local legislation and policies like it, effected a notable transformation in the way the law and society view people with disabilities. For the first time in the nation’s history, people with disabilities are truly recognized in law to be among the We in “We the People”. Laws and regulations that protect people with disabilities’ right to access and use public transportation, sidewalks, and Rights-of-Way also promote their right to be full and equal parts of their communities.

Consequently, it is undeniable that people with disabilities have more entitlements to access public spaces than ever before. However, this progress should be an inspiration for the federal government, states, and localities to make more progress and move consistently and intentionally until, at long last, they fulfill the promise of the ADA and ensure people with disabilities truly can “fully participate in all aspects of society”.

## Data Availability

The Inclusive Public Space Project ends on 31 December 2024, at that time the data will be made available in the Research Data Leeds Repository. Link: https://archive.researchdata.leeds.ac.uk/.
